# Clinical presentation and management patterns of ureteral calculi in pregnancy: a 14-year single-institution retrospective study

**DOI:** 10.3389/fmed.2026.1967019

**Published:** 2026-10-01

**Authors:** Zhipeng Zhou, Lina Xu, Yun Lin, Huaishan Hong

**Affiliations:** 1 Department of Urology, Fuzhou University Affiliated Provincial Hospital, Fuzhou, China; 2 Department of Urology, Fujian Provincial Hospital, Fuzhou, China; 3 Department of Neurosurgery, Fuzhou University Affiliated Provincial Hospital, Fuzhou, China; 4 Department of Neurosurgery, Fujian Provincial Hospital, Fuzhou, China

**Keywords:** pregnancy, ureteral calculi, urolithiasis, conservative management, double-J stent, maternal-fetal outcomes

## Abstract

**Objective:**

This study aimed to describe the clinical presentation, diagnostic pathway, and management patterns of ultrasound-confirmed ureteral calculi during pregnancy at a single institution.

**Methods:**

This study collected, summarized, and analyzed clinical data from 120 pregnant patients diagnosed with ureteral calculi at Fujian Provincial Hospital between March 2010 and March 2024. Primary descriptive outcomes were initial symptom response, documented spontaneous stone passage, subsequent urinary decompression, and residual calculi after pregnancy. Secondary outcomes included technical success of decompression and changes in selected laboratory findings. Maternal-fetal follow-up was not standardized through delivery, and comprehensive obstetric and neonatal outcomes were not systematically available.

**Results:**

All patients were initially managed with conservative treatment and achieved symptomatic relief within 24 h. Spontaneous stone expulsion was documented in 90 patients: 51 at ≤1 month, 17 at >1–3 months, and 22 at >3 months. Urinary decompression was required in 57 patients because of fever, aggravated hydronephrosis, or recurrent renal colic. Retrograde D-J stent placement was attempted in all 57 patients and was successful in 47 (82.5%); the remaining 10 patients underwent percutaneous nephrostomy after unsuccessful stent placement. Cystoscopic D-J stent placement attempts were performed using topical urethral anesthesia with 10% lidocaine, whereas percutaneous nephrostomy was performed under local infiltration anesthesia; no miscarriage or preterm labor temporally associated with urinary decompression or the related anesthesia was documented among the 57 patients who underwent decompression. Post-procedural fetal heart rate monitoring was performed in all 57 patients, and no abnormal findings were documented. Thirty cases retained calculi after completion of pregnancy; among them, 8 underwent postpartum ESWL and 22 underwent postpartum ureteroscopic lithotripsy. Among the 120 analyzed patients, neither ESWL nor ureteroscopic holmium laser lithotripsy (URS-HLL) was performed during pregnancy. Among eight febrile patients, mean white blood cell (WBC) count decreased from 14.9 ± 0.19 × 10^9^/L to 6.7 ± 0.30 × 10^9^/L (paired t-test, *P* < 0.001). Mean procalcitonin (PCT) was 13.86 ± 3.57 ng/mL before treatment, whereas post-treatment values were reported as 0 ng/mL because they fell below the laboratory reporting limit; accordingly, the PCT change was summarized descriptively without inferential testing. Of 38 patients with positive urine leukocytes pretreatment, all tested negative post-treatment (*P* < 0.001). Among 101 cases with pretreatment hematuria, 86 converted to negative after treatment (*P* < 0.001).

**Conclusions:**

This cohort describes a conservative-first management pathway with selective urinary decompression when symptoms or complications persisted. No procedure- or anesthesia-related miscarriage, preterm labor, abnormal post-procedural fetal heart rate finding, or serious urological complication were documented during hospitalization or available urological follow-up among patients who underwent decompression; however, these observations do not establish comprehensive maternal-fetal safety.

## Introduction

Urinary calculi represent a clinically prevalent condition in the field of urology (1). Given the distinctive dietary and physiological conditions during pregnancy, as well as the improvement in living standards in China, the incidence of urinary calculi in pregnancy has shown a progressive annual increase (2). However, the prevalence of ureteral calculi during pregnancy is relatively low. Due to the distinct anatomical location, ureteral calculi often present a different clinical urgency profile compared to renal stones. Ureteral calculi frequently induce renal colic, hydronephrosis, spontaneous renal rupture, severe urinary tract infections, and in severe cases, may lead to urosepsis (3). The aforementioned complications are particularly challenging and hazardous to manage during pregnancy.

Timely and appropriate management of ureteral calculi during pregnancy is critical, as inadequate intervention may seriously compromise both maternal and fetal health (4). The physiological anatomy undergoes alterations during pregnancy primarily due to ureteral compression by the enlarging uterus. Compounded by the limited applicability of imaging modalities during this period, the diagnosis of ureteral calculi is frequently challenging, potentially leading to deviations in therapeutic management (5). Pain attributable to ureteral calculi during pregnancy is frequently misdiagnosed as appendicitis, diverticulitis, or placental abruption (6). Meanwhile, as a recognized safe and effective diagnostic modality during pregnancy, ultrasonography frequently fails to detect ureteral calculi in pregnant patients due to interference from an enlarged uterus, intestinal gas, or radiolucent stones (7). This presents considerable challenges for diagnosis and treatment in the field of urology. Severe pain induced by ureteral calculi may precipitate miscarriage or premature labor in pregnant women. Therefore, effective analgesia is crucial for patients with concurrent pregnancy and ureteral calculi.

Additionally, it is imperative to accord due attention to asymptomatic ureteral calculi during pregnancy. Hydronephrosis resulting from small or negatively identified ureteral calculi is frequently misdiagnosed during ultrasonographic examination as being caused by ureteral compression from an enlarged uterus (8). As complications manifest and the pregnancy progresses, the management becomes increasingly challenging. Evidence in this population remains predominantly observational, and practice varies according to disease severity, gestational age, available expertise, and institutional resources. We therefore reviewed our 14-year experience to describe the clinical presentation, ultrasound-based diagnostic pathway, management patterns, and recorded urological outcomes of pregnant patients with ureteral calculi. The study was not designed to compare treatment efficacy or provide a comprehensive assessment of maternal, fetal, or neonatal safety.

## Material and methods

### Study design and patients

This study retrospectively analyzed data from 120 patients diagnosed with pregnancy-associated ureteral calculi who were admitted to Fujian Provincial Hospital between March 2010 and March 2024. The dataset was assembled from an archived institutional inpatient registry. A small institutional case series of ureteroscopic holmium laser lithotripsy (URS-HLL) during pregnancy was maintained separately and was not incorporated into this registry; those patients were therefore outside the source dataset rather than excluded after eligibility assessment. This study was conducted at Fujian Provincial Hospital and approved by its Ethics Committee (Approval No. K2024-11-013). Written informed consent was obtained from all participants prior to their involvement. All subjects underwent renal ultrasonography to confirm the presence of ureteral calculi. Inclusion criteria were pregnancy at diagnosis, age ≥18 years, direct ultrasonographic visualization of a ureteral calculus, available laboratory data, and available urological follow-up. Exclusion criteria were non-ureteral calculi, absent laboratory data, loss to urological follow-up after diagnosis, or strong clinical suspicion without direct ultrasonographic confirmation. The archived dataset contained the final eligible cohort but did not retain the initial screening denominator or reason-specific exclusion counts The recruitment procedure for study participants is delineated in Figure 1.

**Figure 1:**
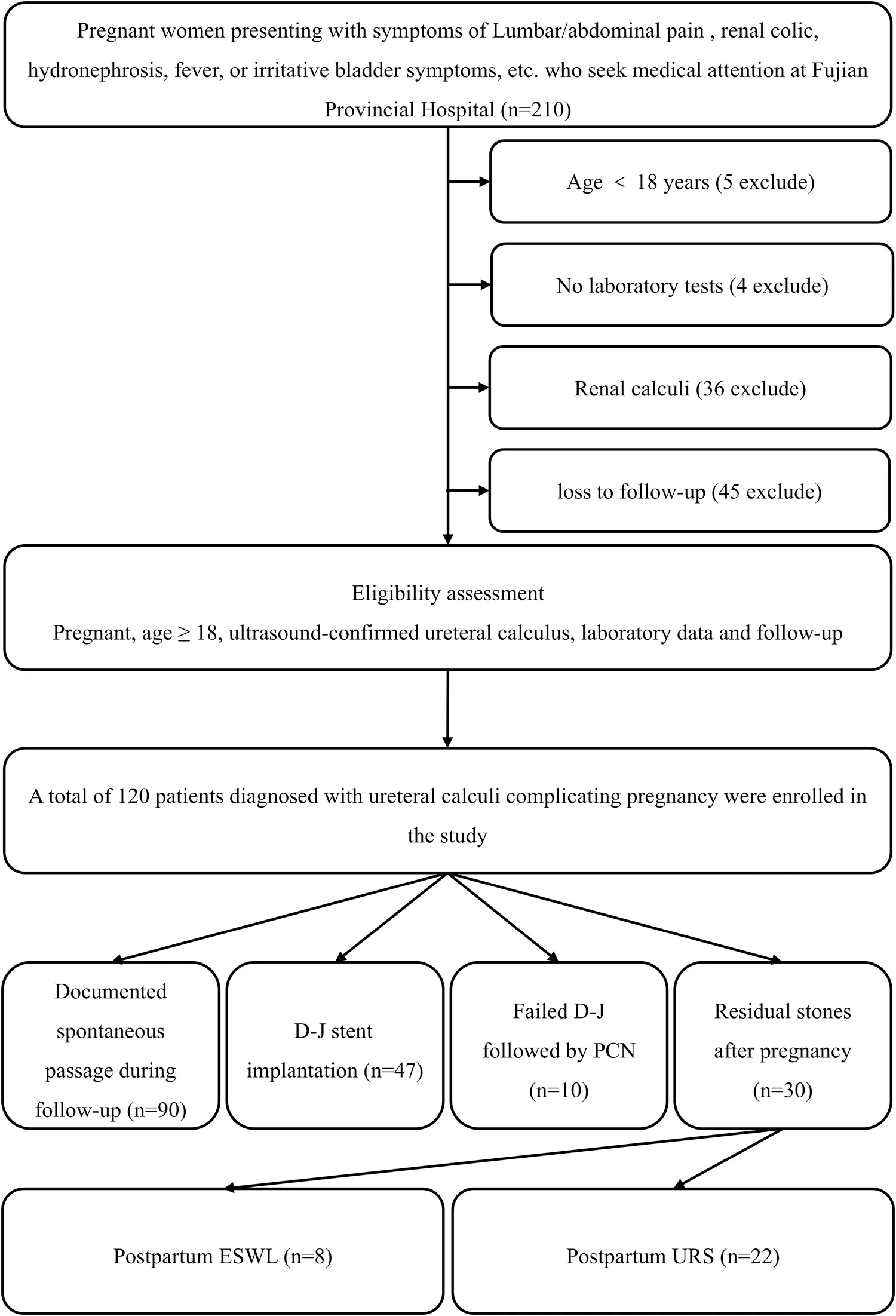
Flowchart of recoverable patient selection information, management, and outcomes. D-J, double-J; PCN, percutaneous nephrostomy; ESWL, extracorporeal shock wave lithotripsy; URS, ureteroscopy.

### Diagnostic assessment

All patients underwent urinary tract ultrasonography. Neither kidney-ureter-bladder radiography, computed tomography urography, nor magnetic resonance urography was performed during pregnancy. Direct visualization of a ureteral calculus was required for inclusion; hydronephrosis alone was not accepted as evidence of pathological obstruction, thereby reducing misclassification of physiological gestational hydronephrosis. Renal sinus separation was categorized as mild (1-2 cm), moderate (2-3 cm), or severe (>3 cm). Because the study spanned 14 years, ultrasound acquisition and reporting were not governed by a single prospective protocol, and Doppler indices or transvaginal ultrasound details were not consistently available.

### Outcome definitions and data availability

The primary descriptive outcomes were initial symptom response (documented improvement in pain within 24 h), documented spontaneous stone passage and its recorded timing, subsequent need and type of urinary decompression, and residual calculi identified after pregnancy. Secondary outcomes were technical success of D-J stent placement, serious urological treatment-related complications documented during hospitalization or available urological follow-up, paired changes in blood white blood cell count and procalcitonin among febrile patients, and changes in urinalysis. Stone passage and residual calculi were abstracted as documented in the treating records and follow-up imaging; a uniform computed tomography-based stone-free definition was not used during pregnancy. Miscarriage or preterm labor after urinary decompression was specifically reviewed when documented during hospitalization or available urological follow-up. Post-procedural fetal heart rate findings were reviewed for all patients undergoing urinary decompression. The duration of maternal-fetal follow-up was not standardized, and follow-up through delivery could not be confirmed for all patients. Accordingly, miscarriage, preterm delivery, fetal loss, other obstetric complications, and neonatal complications were not systematically recorded through delivery and were not analyzed as comprehensive safety outcomes.

### Treatment protocols

All patients initially received conservative management comprising clinical observation, hydration, infection control when indicated, and obstetric evaluation. In patients with renal colic, progesterone was administered at the obstetrician's discretion as an adjunct intended to reduce uterine irritability associated with pain rather than as a direct analgesic. Progesterone 20 mg was administered intramuscularly once or twice daily, according to symptom severity, for 3−5 days or until clinically meaningful symptom relief. In selected patients with mild colic, oral micronized progesterone (100-200 mg/day) was used as an alternative. When additional analgesia was required, bucinnazine or pethidine was administered after joint urological and obstetric assessment. A cephalosporin or penicillin was prescribed for concomitant infection. Escalation to urinary decompression was considered for persistent or recurrent renal colic, fever or worsening infection, or progressive hydronephrosis despite conservative management. Treatment was selected by the attending urologist in consultation with obstetric services according to the clinical condition and technical feasibility; allocation was not randomized. A 4.7-Fr, 26-cm D-J ureteral stent (Bard Medical, Covington, GA, USA) was inserted under cystoscopic guidance using topical urethral anesthesia with 10% lidocaine, with ultrasonography used to confirm positioning. Percutaneous nephrostomy was performed under local infiltration anesthesia when retrograde stent placement was unsuccessful. These anesthetic approaches were selected to minimize systemic anesthetic exposure. Fetal heart rate monitoring was performed after each decompressive procedure.

Urinary tract ultrasonography was performed monthly during pregnancy for routine urological follow-up. A D-J stent was scheduled for removal after documented stone passage and, when prolonged drainage was required, replacement every 1−3 months was recommended because pregnancy-associated hypercalciuria, hyperuricosuria, and urinary stasis increase the risk of encrustation. Nephrostomy tubes were exchanged approximately monthly when continued drainage was required. Definitive stone treatment was deferred until after pregnancy; ESWL was strictly avoided because pregnancy is an absolute contraindication. Although post-procedural fetal heart rate assessment was documented, the exact gestational timing of individual interventions and standardized details of continuous intra-procedural fetal monitoring were not consistently captured in the archived records and therefore could not be analyzed.

### Statistical analysis

Continuous variables are presented as mean ± standard deviation or median [interquartile range (IQR)], as appropriate, and categorical variables as number and percentage. Paired t-tests were used for the archived paired pre- and post-treatment white blood cell measurements; formal normality-test output was not retained, and the small febrile subgroup requires cautious interpretation. No inferential test was reported for procalcitonin because exact post-treatment values were censored below the laboratory reporting limit. McNemar's test was used for paired categorical urinalysis data. Missing data were handled by complete-case analysis, no imputation was performed, and outcome-specific denominators are reported. Because this was a descriptive study with nonrandom treatment selection, no between-treatment comparative analysis or multivariable adjustment was performed. Analyses were conducted using SPSS version 23.0 (IBM Corporation, Armonk, NY, USA), with two-sided *P* < 0.05 considered statistically significant.

## Results

### Demographic characteristics of patients

This study enrolled a total of 120 pregnant patients diagnosed with ureteral calculi. The mean age of the participants was 25 years [interquartile range (IQR): 23–27], with a mean gestational age of 25 weeks (IQR: 22–36). The average stone size was 7 mm (IQR: 7–8) by 8 mm (IQR: 5–10). Among the subjects, 11 were in the first trimester, 64 in the second trimester, and 45 in the last trimester (Table 1). Localization of calculi included 47 cases in the left ureter, 67 in the right ureter, and 6 bilateral cases. Based on ureteral segment involvement, there were 60 cases in the upper segment, 35 in the middle segment, and 25 in the lower segment. A total of 116 patients presented with lumbago and abdominal pain, 58 exhibited nausea and vomiting, 19 demonstrated gross hematuria, and 12 displayed bladder irritation symptoms including urinary frequency, urgency, and dysuria. Eight patients experienced pyrexia, while four were asymptomatic and were diagnosed with ureteral calculi solely during prenatal examinations. Hydronephrosis was observed in 106 patients, among whom 62 cases were classified as mild, 44 as moderate, and no severe cases were recorded (Table 2).

**Table 1:** Baseline demographic and stone characteristics of the study population (*n* = 120).

Characteristic	Value
Age at diagnosis, years	25 (23–27)
Gestational age at diagnosis, weeks	25 (22–36)
Stone dimension 1, mm	7 (7–8)
Stone dimension 2, mm	8 (5–10)
First trimester	11 (9.2%)
Second trimester	64 (53.3%)
Third trimester	45 (37.5%)

Data are median (IQR) or *n* (%).

**Table 2:** Clinical presentation and imaging characteristics (*n* = 120).

Characteristic	*n* (%)
Left ureter	47 (39.2)
Right ureter	67 (55.8)
Bilateral ureters	6 (5.0)
Upper ureter	60 (50.0)
Middle ureter	35 (29.2)
Lower ureter	25 (20.8)
Lumbar or abdominal pain	116 (96.7)
Nausea and vomiting	58 (48.3)
Gross hematuria	19 (15.8)
Bladder irritation symptoms	12 (10.0)
Fever	8 (6.7)
Asymptomatic	4 (3.3)
Hydronephrosis	106 (88.3)
Mild	62 (51.7)
Moderate	44 (36.7)
Severe	0 (0.0)

Data are showed as *n* (%) for counts.

### Treatment outcomes

All patients had documented initial symptom improvement within 24 h after conservative management; this early response was not considered equivalent to definitive treatment success because symptoms or complications subsequently prompted intervention in some patients. During follow-up, 90 patients had documented spontaneous stone passage: 51 at ≤1 month, 17 at >1–3 months, and 22 at >3 months. D-J stent placement was attempted in 57 patients because of fever, progressive hydronephrosis, persistent or recurrent renal colic, or worsening infection. Placement was successful in 47 patients; the remaining 10 patients underwent percutaneous nephrostomy after unsuccessful stenting. Symptomatic improvement after decompression was documented, and no serious urological complication such as severe encrustation, an unretrievable stent, or sepsis was recorded during hospitalization or available urological follow-up. Thirty patients had residual calculi after pregnancy; 8 underwent postpartum ESWL and 22 underwent postpartum ureteroscopic lithotripsy. Among the 120 analyzed patients, neither ESWL nor ureteroscopic holmium laser lithotripsy (URS-HLL) was performed during pregnancy (Table 3). Cystoscopic D-J stent placement was attempted using topical urethral anesthesia with 10% lidocaine, whereas percutaneous nephrostomy was performed under local infiltration anesthesia. No miscarriage or preterm labor temporally associated with urinary decompression or the related anesthesia was documented during hospitalization or available follow-up among the 57 patients who required decompression. Post-procedural fetal heart rate monitoring was performed in all 57 patients who required decompression, and no abnormal findings were documented. The duration of maternal-fetal follow-up was not standardized, and follow-up through delivery was not documented for all patients; therefore, fetal loss, preterm delivery, other obstetric complications, and neonatal complications could not be comprehensively assessed.

**Table 3:** Management and treatment outcomes of ureteral calculi during pregnancy.

Management or outcome	*n* (%)
Initial conservative management	120 (100.0)
Initial symptom improvement within 24 h	120 (100.0)
Spontaneous stone passage during follow-up	90 (75.0)
Within 1 month	51 (42.5)
> 1 to 3 months	17 (14.2)
> 3 months	22 (18.3)
Successful D-J stent placement	47 (39.2)
Failed D-J placement followed by PCN	10 (8.3)
Serious urological complication documented	0 (0.0)
Residual stones after pregnancy	30 (25.0)
Postpartum ESWL	8 (6.7)
Postpartum ureteroscopic lithotripsy	22 (18.3)
ESWL or ureteroscopic lithotripsy during pregnancy	0 (0.0)

Data are presented as *n* (%) f. PCN, percutaneous nephrostomy; ESWL, extracorporeal shock wave lithotripsy.

### Comparison of laboratory findings of patients

The white blood count (WBC) and procalcitonin (PCT) levels in the blood of eight febrile patients prior to treatment were 14.9 ± 0.19 × 10^9^/L and 13.86 ± 3.57 ng/mL, respectively. Following treatment, the mean WBC count decreased to 6.7 ± 0.30 × 10^9^/L (paired t-test, *P* < 0.001). The post-treatment PCT value was reported as 0 ng/mL because it was below the laboratory reporting limit; accordingly, no inferential test was performed for PCT (Table 4).

**Table 4:** Comparison of blood WBC count and PCT level before and after treatment in febrile patients (*n* = 8).

Variable	Before treatment	After treatment	*P*-value
WBC, × 10⁹/L	14.9 ± 0.19	6.7 ± 0.30	<0.001
PCT, ng/mL	13.86 ± 3.57	Below reporting limit	<0.001

Data are mean ± SD. The value of PCT is below reporting limit, so it is calculated as 0. WBC, white blood cell; PCT, procalcitonin.

The urinary parameters demonstrated significant improvement following the treatment. Among the 38 patients who presented with positive urinary WBC counts at baseline, all cases (100%) achieved conversion to negative status post-treatment (McNemar's exact test, *P* < 0.001). Regarding urinary erythrocytes (RBC), 101 patients tested positive prior to treatment, among whom 86 (85.15%) exhibited negative conversion following treatment. McNemar's test demonstrated a statistically significant reduction in the prevalence of hematuria (*χ*^2^= 84.01, *P* < 0.001). These results indicate that the treatment is highly effective in eliminating urinary inflammation and occult blood in patients with calculi (Table 5).

**Table 5:** Comparison of positive urinary WBC and RBC findings before and after treatment (*n* = 120).

Urinary parameter	Before treatment, *n* (%)	After treatment, *n* (%)	*P*-value
Positive urinary WBC	38 (31.7)	0 (0.0)	<0.001
Positive urinary RBC	101 (84.2)	15 (12.5)	<0.001

*P*-values were calculated using McNemar's test. WBC, white blood cell; RBC, red blood cell.

## Discussion

The incidence of urinary tract calculi during pregnancy varies depending on geographic location and population demographics, with existing literature reporting a prevalence rate ranging from 0.03% to 0.3% (9). Urinary calculi in pregnant women are frequently induced by multiple factors including dietary habits, physical activity levels, metabolic adaptations, and physiological structural changes, typically manifesting during the second and third trimesters of gestation (10, 11). In most cases, smaller calculi can be expelled spontaneously or facilitated by expulsion-inducing medications combined with physical activity (12). However, during pregnancy, the expulsion of stones is obstructed due to constraints in the use of lithotriptic medications, restricted capacity for strenuous physical activity, and ureteral compression. Consequently, even small calculi may be unable to pass spontaneously. Therefore, patients presenting with ureteral calculi during pregnancy frequently exhibit symptoms including renal colic, hydronephrosis, abdominal pain, hematuria, and urinary tract infections, with some cases also manifesting concomitant nausea and vomiting (13–15). This study identified that among 120 patients with ureteral calculi complicating pregnancy, 96.7% presented with lumbar/abdominal pain, 84.2% exhibited hematuria, 88.3% manifested hydronephrosis, and 48.3% were accompanied by nausea and vomiting. These findings are consistent with the previously documented literature. It is particularly important to note that uncomplicated ureteral calculi do not pose specific adverse effects on pregnant women or the fetus. However, severe renal colic, hydronephrosis, or urinary tract infections induced by the calculi may precipitate preterm labor or miscarriage (16). Therefore, managing the complications is more critical than addressing the calculi themselves.

Ultrasonography represents the first-line diagnostic modality for ureteral calculi in pregnancy. Ultrasonography is not only radiation-free and considered safe for pregnant women, but it is also capable of detecting ureteral stones with a diameter exceeding 2 mm. Additionally, it can accurately assess the degree of ureteral dilation and the presence of hydronephrosis. However, ultrasound technology also presents certain limitations. During the second and third trimesters of pregnancy, detection of calculi located in the mid-to-distal ureter is often challenging due to interference from the enlarged uterus or intestinal gas, which places greater demands on the technical proficiency and experience of sonographers. Clinicians must also perform comprehensive assessments by integrating clinical symptoms, physical signs, and ancillary diagnostic evaluations (17). Abdominal computed tomography (CT) demonstrates significantly higher sensitivity and accuracy in the detection of calculi compared to ultrasonography (18). Given the radiation risks associated with CT scans to the fetus, particularly during early pregnancy, this diagnostic modality is not routinely employed in the clinical management of ureteral calculosis in pregnant patients (19). While magnetic resonance imaging (MRI) carries no risk of radiation exposure, its utility is limited in the differentiation of calculi due to low sensitivity. Consequently, it is not considered a routine diagnostic modality for stone detection (20). In this study, all 120 patients were diagnosed with ureteral calculi through ultrasonography, and none underwent CT or MRI examinations of the urinary system. Consequently, the findings of this study are generalizable to ultrasound-detectable ureteral calculi in pregnancy, which represents the predominant clinical scenario.

The fundamental principle for managing ureteral calculi during pregnancy is to ensure maternal and fetal safety while preventing any harm to the fetus. The treatment objectives encompass relieving obstruction, alleviating pain, controlling infection, and preserving renal function (17). The literatures indicated that conservative management for ureteral calculi in pregnancy achieved favorable clinical outcomes, with a spontaneous calculi expulsion rate of 70% to 80% (21). In the present study, spontaneous stone passage was documented in 90 patients (75.0%): 51 at ≤1 month, 17 at >1–3 months, and 22 at >3 months, consistent with previously published reports. Viewed in the context of recent evidence, this finding supports conservative management as the initial strategy for clinically stable patients without infection, refractory pain, or progressive obstruction. Nevertheless, the subsequent need for decompression in 57 patients shows that early symptom relief should not be equated with definitive treatment success and underscores the importance of close reassessment. Conservative management is the primary therapeutic approach for ureteral calculi in pregnancy, involving spasmolysis, analgesia, antimicrobial therapy, and fluid supplementation (22). Asymptomatic ureteral calculi may be managed through active surveillance with regular follow-up evaluations. Most patients can safely complete the gestational period under such monitoring, with definitive stone intervention deferred until postpartum. In cases presenting with fever, recurrent renal colic, severe urinary tract infection, solitary kidney, bilateral ureteral stones, or progressively worsening hydronephrosis, priority should be given to surgical interventions such as ureteral D-J stent placement under cystoscopy, percutaneous nephrostomy, or flexible ureteroscopic laser lithotripsy to relieve the obstruction (23–25). In our cohort, cystoscopic D-J stent placement was the most used interventional approach when conservative management failed, particularly for patients requiring urgent decompression or those unsuitable for definitive ureteroscopic lithotripsy (26). A principal advantage of this approach lies in its capacity to effectively relieve obstruction in a minimally invasive manner, thereby alleviating renal colic and hydronephrosis while facilitating infection control. However, the indwelling of DJ stent is associated with complications such as urinary frequency, urgency, dysuria, hematuria, and lumbar/abdominal discomfort. Additionally, given that pregnant women frequently present with concurrent hypercalcemia and hyperuricemia, these conditions predispose to encrustation of the D-J stent, thereby complicating or preventing its removal or replacement. Therefore, it is recommended that the D-J stent be replaced every 1 to 3 months. In cases where patients exhibit extreme intolerance to stent placement, or when ureteral calculi impaction or significant ureteral tortuosity prevents successful D-J stent insertion, ultrasound-guided percutaneous nephrostomy may be considered as an alternative approach to alleviate urinary obstruction (27). Although ultrasound-guided percutaneous nephrostomy can rapidly alleviate renal colic and relieve obstruction, the nephrostomy tube is prone to displacement and detachment, requiring intensive nursing care. It is also associated with a high risk of complications such as catheter-related infections and bleeding. Consequently, this procedure is generally not considered the first-line treatment, but rather an alternative approach when retrograde D-J stent placement fails. Furthermore, routine replacement of the nephrostomy tubes every month is recommended. Our findings are also consistent with recent reviews indicating that D-J stenting and percutaneous nephrostomy remain appropriate for urgent decompression when definitive ureteroscopy is unavailable, unsuitable, or cannot be performed promptly, although device-related morbidity and repeated exchanges remain important disadvantages during pregnancy.

With advancements in ureteroscopic techniques, particularly the refinement of flexible ureteroscopy (URS) and equipment innovations, the surgical management of ureteral calculi during pregnancy no longer entails the extensive contraindications observed in the past. A recent systematic review and meta-analysis by Jin et al. (24) comparing URS versus internal ureteral stents in 789 pregnant women with urolithiasis found that URS achieved a pooled stone-free rate of 91% and an operative success rate of 99%. Overall complications after URS were approximately 1%, and serious complications (Clavien-Dindo III-V) were <1%. Normal fertility outcomes were 99% for both URS and stenting, but URS provided definitive stone clearance without the need for repeated stent exchanges or the risk of stent encrustation.

The 2025 European Association of Urology (EAU) guidelines on urolithiasis now recommend ureteroscopic laser lithotripsy as a safe and effective first-line option when conservative management fails during pregnancy, particularly for patients with persistent symptoms or obstruction who are candidates for definitive treatment (17). A large multicenter study by Juliebø-Jones et al. (10) on behalf of the EAU Section of Endourology further confirmed the safety and efficacy of URS in pregnancy across multiple institutions. Optimal outcomes depend on a multidisciplinary collaborative effort involving urology, anesthesiology, obstetrics, and neonatology (28–30). While historical concerns about the long-term effects of anesthesia on fetal development have been raised, current evidence from large cohort studies and meta-analyses has not demonstrated an increased risk of adverse pregnancy outcomes with modern anesthetic techniques (24, 28). Taken together, current recommendations support a tiered rather than uniform treatment strategy: conservative care for uncomplicated cases, prompt decompression when complications develop, and definitive URS as a reasonable alternative for appropriately selected patients in experienced multidisciplinary centers.

None of the 120 analyzed patients underwent ureteroscopic lithotripsy or ESWL during pregnancy. Thus, the present data cannot evaluate definitive ureteroscopic treatment during pregnancy, and the discussion of URS is based solely on published evidence rather than procedures performed in this cohort. Although the study covered 14 years, the archived dataset did not contain sufficiently standardized protocol-era variables to support a reliable period-stratified analysis. Changes in imaging equipment, anesthesia practice, clinical guidance, and multidisciplinary care over time therefore remain potential sources of heterogeneity.

Any URS-HLL procedures performed at our institution during the broader study period belonged to the separately maintained surgical series described in the Methods and were not part of the 120-patient cohort; no outcomes from those cases are reported or interpreted in the present study.

The present findings are best interpreted as a description of one institution's management pathway. Initial symptom improvement was documented in all patients, but 57 later required decompression because of recurrent or progressive clinical problems. Documented spontaneous passage occurred in 90 patients, while 30 had residual calculi after pregnancy and received postpartum ESWL or ureteroscopic lithotripsy. No serious urological complication related to drainage was documented during the available follow-up. All decompressive procedures used topical urethral or local infiltration anesthesia, and no miscarriage or preterm labor temporally associated with the procedures or anesthesia was documented among these 57 patients. Post-procedural fetal heart rate monitoring was documented in all 57 patients, with no abnormal findings. However, although procedure-associated miscarriage and preterm labor were reviewed from the available records, maternal-fetal follow-up was not standardized in duration or consistently continued through delivery; fetal loss, preterm delivery, other obstetric complications, and neonatal outcomes were not systematically captured. The present data therefore support only the absence of documented treatment-related maternal-fetal adverse events during hospitalization or available urological follow-up and should not be interpreted as evidence of comprehensive maternal-fetal safety.

In contrast, URS-HLL offers the advantage of definitive stone clearance in a single procedure, eliminating the need for repeated interventions and the associated risks and patient burden. A single-center study by Gull et al. (29) reported excellent outcomes with URS-HLL in pregnancy, with a 95% stone-free rate and no major complications. For patients with persistent symptoms, progressive hydronephrosis, or infection despite conservative management, URS-HLL should be considered as a first-line interventional option in centers with appropriate expertise and multidisciplinary support. Thulium fiber laser lithotripsy, which offers superior stone ablation efficiency and reduced retropulsion compared to holmium laser, has also been successfully used in pregnant patients as an alternative technology.

This study has several limitations. Its retrospective, single-center design introduces selection bias, information bias, confounding by indication, and limited generalizability. The sample size and number of febrile or invasively treated patients were modest. The initial screening denominator and reason-specific exclusions were not retained, and ultrasound protocols were not uniform across the 14-year period. Although the anesthetic techniques and post-procedural fetal heart rate assessments were available, maternal-fetal follow-up was not standardized through delivery, and intervention timing by gestational age, continuous intra-procedural fetal monitoring, fetal distress beyond the recorded post-procedural assessments, fetal loss, preterm delivery, other obstetric complications, and neonatal outcomes were not systematically available. Missing variables were not imputed. The study did not include comparative treatment groups, multivariable adjustment, or a standardized computed tomography-based stone-free endpoint, and it could not evaluate temporal changes in practice. Because the separately maintained URS-HLL series was not incorporated into the archived registry, the present study also could not evaluate ureteroscopic treatment during pregnancy.

Future research should include prospective or matched retrospective studies comparing conservative/decompressive strategies with definitive ureteroscopic lithotripsy in pregnant patients with ureteral calculi. Such studies should evaluate not only clinical outcomes but also patient-reported quality of life, healthcare resource utilization, and long-term maternal and fetal outcomes.

## Conclusions

This 14-year single-institution cohort describes the presentation and management of ultrasound-confirmed ureteral calculi during pregnancy. Conservative management was used initially, and selective D-J stenting or percutaneous nephrostomy provided urinary decompression when symptoms or complications persisted. Among patients undergoing decompression, no procedure- or anesthesia-related miscarriage, preterm labor, abnormal post-procedural fetal heart rate finding, or serious urological complication were documented during hospitalization or available urological follow-up. Because maternal-fetal follow-up was not standardized through delivery, these observations indicate only an absence of documented treatment-related adverse events during the available follow-up and do not establish comparative effectiveness or comprehensive maternal-fetal safety. Treatment should remain individualized, multidisciplinary, and aligned with current recommendations; URS may be considered in experienced centers, whereas ESWL is contraindicated during pregnancy.

## Data Availability

The raw data supporting the conclusions of this article will be made available by the authors, without undue reservation.
